# Expression Patterns of SMAD1–8 in the Peripheral Facial Nerve Following Compressive Nerve Injury or Axotomy

**DOI:** 10.3390/ijms26052291

**Published:** 2025-03-04

**Authors:** Jae Min Lee, Dong Keon Yon, Sung Soo Kim, Seung Geun Yeo

**Affiliations:** 1 Department of Otorhinolaryngology–Head and Neck Surgery, College of Medicine, Kyung Hee University Medical Center, Kyung Hee University, Seoul 02447, Republic of Korea; sunjaesa@hanmail.net; 2 Center for Digital Health, Medical Science Research Institute, Kyung Hee University School of Medicine, Kyung Hee University Medical Center, Seoul 02447, Republic of Korea; yondg@khu.ac.kr; 3 Department of Biochemistry and Molecular Biology, College of Medicine, Kyung Hee University, Seoul 02447, Republic of Korea; sgskim@khu.ac.kr; 4 Department of Convergence Medicine, College of Medicine, Kyung Hee University, Seoul 02447, Republic of Korea

**Keywords:** facial nerve injury, compression, axotomy, Suppressor of mothers against decapentaplegic homolog (SMAD)

## Abstract

Facial nerve injury can lead to significant functional impairment, emotional impacts, and difficulties in social and economic activities. Although peripheral nerves have the potential for recovery, incomplete regeneration can pose challenges. Suppressor of Mothers Against Decapentaplegic Homolog (SMAD) proteins are crucial in the nerve-regeneration process. The study aimed to investigate the changes in SMAD protein expression involved in peripheral nerve regeneration following facial nerve injury induced by compression or axotomy in a pre-clinical study conducted on Sprague Dawley rats. Facial nerve recovery was assessed at 1, 2, 3, 4, 8, and 12 weeks post-facial nerve compression and axotomy using behavioral tests, including whisker movement and eyelid blink-reflex tests. Additionally, the role of SMAD proteins in the nerve regeneration process was evaluated by analyzing the expression of SMAD1–8 proteins at 2 and 12 weeks post-injury. Behavioral tests revealed significant impairment in facial nerve function in both the Compression and Axotomy groups compared with the Sham group at early time points. Recovery was observed in the Compression group by 2 weeks, whereas the Axotomy group exhibited prolonged impairment through 12 weeks. SMAD protein analyses showed increased expression of SMAD2, SMAD7, and SMAD8 following compression injury, whereas axotomy led to more extensive increases in expression that included SMAD1, SMAD2, SMAD3, SMAD4, SMAD6, SMAD7, and SMAD8. These findings suggest that SMAD proteins play differential roles in nerve regeneration following facial nerve injuries caused by compression versus axotomy. The distinct expression patterns of SMAD proteins highlight their potential as therapeutic targets for enhancing nerve regeneration and functional recovery in peripheral nerve injuries.

## 1. Introduction

Facial nerve injury is a significant clinical condition that can lead to considerable functional impairments and profoundly affect a patient’s quality of life. Such injuries can result from various causes, including trauma, surgical procedures and infections, leading to symptoms such as muscle weakness, twitching, or paralysis on one side of the face. The psychological and social implications of facial nerve dysfunction can be severe, affecting self-esteem and interpersonal interactions [1]. Peripheral facial nerve injury can result in a range of clinical signs and symptoms, depending on the severity and location of the damage [2]. These may include facial asymmetry, drooping of the mouth, loss of the nasolabial fold, altered taste sensation, hyperacusis, lacrimal dysfunction, and difficulties with fine motor control of facial muscles [3]. Typically, the central nervous system (CNS), which includes the brain and spinal cord, does not regenerate damaged axons following physical injury, often leading to permanent disabilities. In contrast, the peripheral nervous system (PNS), which comprises peripheral nerves that innervate the body, can activate a spontaneous regeneration program, allowing severed axons to often heal and navigate back to their original targets. This regenerative capability of the PNS does not necessarily require the nerve cell body to be within the CNS but rather relies on intrinsic mechanisms and the crucial role of supporting structures like Schwann cells [4]. In fact, peripheral nerve injuries that are not severe, such as those involving nerve compression, often achieve near-complete functional recovery over time [5]. However, severe nerve injuries caused by axotomy, while capable of axonal degeneration and regeneration, may not always lead to full functional recovery [6].

Peripheral nerve compression injuries that do not cause complete transection typically result in localized demyelination and mild axonal damage [5]. Since the nerve structure remains largely intact, the regeneration process can be simpler. Schwann cells, which are essential for nerve repair, rapidly remyelinate demyelinated axons, a process crucial for restoring the insulation of nerve fibers and ensuring efficient signal transmission [7]. Schwann cells respond quickly to injury by promoting remyelination and axonal growth, thereby helping to restore function. These factors contribute to rapid functional recovery and successful nerve repair following nerve compression injuries. In contrast, following nerve axotomy, the distal portion of the axon undergoes Wallerian degeneration, during which both the axon and myelin sheath degrade distal to the injury site [8]. Proximally, Schwann cells proliferate and migrate to the transected fiber, forming Bands of Büngner, which provide support and pathways for reconnection [7]. Following axotomy, the nerve cell body increases the synthesis of RNA, proteins, and enzymes necessary for axonal growth and repair, thereby promoting axonal regeneration [9].

Not all causes of facial paralysis are understood, but known causes include Bell’s palsy, infections, trauma, tumors, congenital factors, recurrent factors, metabolic issues, and systemic diseases [2]. Clinical treatments for peripheral nerve damage encompass a range of strategies, including drug therapy with anti-inflammatory agents such as steroids, surgical interventions to repair or reconstruct nerve pathways, and emerging approaches like stem cell therapy that aim to enhance nerve regeneration and functional recovery [10]. Steroid therapy is effective in reducing inflammation, swelling, and immune responses. However, long-term use can lead to side effects such as osteoporosis, diabetes, hypertension, and an increased risk of infection [11]. Surgical interventions for peripheral nerve injuries, such as direct suturing, autologous nerve grafting, and nerve substitution techniques, are standard practices. However, these procedures can lead to undesirable outcomes, including sensory loss, scarring, and neuroma formation at the donor site. Moreover, they frequently fall short of completely restoring nerve function, underscoring the necessity for enhanced surgical techniques and complementary therapies [12]. Stem cell therapy, utilizing Schwann cells and mesenchymal stem cells, presents numerous advantages in peripheral nerve regeneration. These include promoting nerve regeneration, offering neuroprotection, reducing inflammation, modulating immune responses, encouraging angiogenesis, and aiding in tissue repair. However, this therapy also faces significant challenges, such as the potential for neuropathic pain, immune reactions, tumor formation, infection risks, and high costs [13]. Ultimately, there are no complete treatments that ensure full functional recovery.

SMAD (Suppressor of Mothers Against Decapentaplegic Homolog) proteins, including SMAD1–8, are crucial components of signaling pathways that play essential roles in various cellular processes, such as nerve regeneration, growth, and repair. These roles underscore the significant importance of this protein family in facilitating recovery following peripheral nerve injury [14]. SMAD proteins are central mediators of the transforming growth factor-beta (TGF-β) signaling pathway. This pathway plays a pivotal role in regulating cell proliferation, differentiation, and survival, highlighting its importance in various physiological and pathological processes [15]. SMAD1/5/8 proteins are receptor-regulated SMADs that play a crucial role in bone morphogenetic protein (BMP) signaling. This pathway is instrumental in promoting nerve differentiation and repair, thereby contributing significantly to the development of the nervous system [16]. These pathways influence the growth and differentiation of Schwann cells, which are essential for nerve repair and remyelination. The primary SMAD signaling pathways for nerve regeneration are the BMP/SMAD pathway and the PI3K/GSK3/SMAD1 pathway [14]. Studies on peripheral nerve regeneration and degeneration have shown that SMAD expression can increase or decrease depending on the type of nerve, method of injury, and severity. The study aimed to explore the behavioral aspects related to nerve regeneration using compression and axotomy in a facial nerve injury animal model and to investigate the expression of SMAD1–8 proteins following peripheral facial nerve injury in a pre-clinical animal study conducted on Sprague Dawley rats.

## 2. Results

### 2.1. Evaluation of Facial Nerve Injury Recovery Using Whisker Movement and Eyelid Blink-Reflex Tests

**Figure 1:**
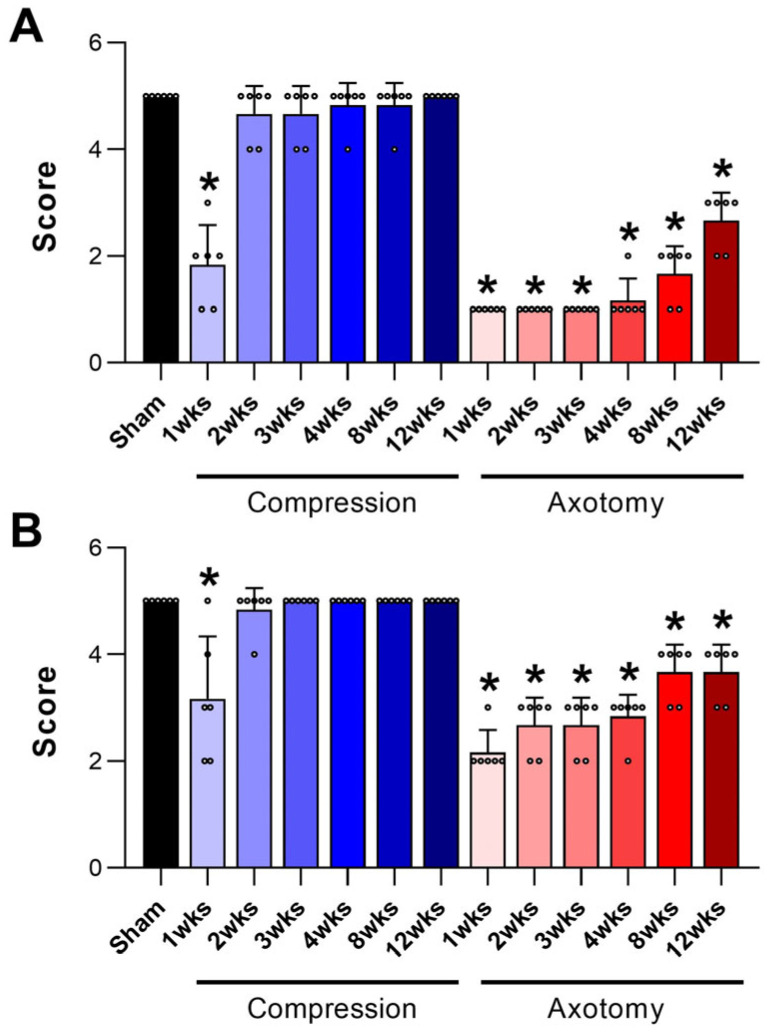
Evaluation of facial nerve functional recovery using behavioral tests following nerve injury. (**A**) Whisker movement (vibrissae muscle) test results at 1, 2, 3, 4, 8, and 12 weeks post injury, showing significantly reduced scores in both Compression and Axotomy groups compared with the Sham group at 1 week. Recovery in the Compression group is indicated by comparable scores to the Sham group from 3 weeks onwards, while the Axotomy group continued to show impaired function through 12 weeks. (**B**) Eyelid blink-reflex test results, displaying significantly lower scores in both injury groups at 1 week compared with the Sham group. Similar to whisker movement test results, the Compression group recovered by 1 weeks but the Axotomy group did not, exhibiting persistent deficits through 12 weeks. Data are presented as means ± S.D., with a sample size of *n* = 6 per group (* *p* < 0.05 vs. Sham group). Abbreviations: wks, weeks.

The recovery of facial nerve function was assessed using whisker movement and eyelid blink-reflex tests at 1, 2, 3, 4, 8, and 12 weeks post-injury. These tests were conducted on male Sprague-Dawley rats, as described in the methods section, where details of their housing, acclimatization, and group assignments (Compression, Axotomy, or Sham) are provided. These behavioral assessments are critical for evaluating the functional recovery of the facial nerve, and the results, presented in Figure 1, highlight the recovery patterns observed across different experimental groups. These tests are useful for directly evaluating the completeness of functional recovery of the facial nerve. In the whisker movement (vibrissae muscle) test (Figure 1A), whisker movement scores were significantly lower in both the Compression and Axotomy groups compared with the Sham group at 1 week (F = 311.235, *p* < 0.001) after facial nerve injury. Similar to the results of eyelid blink tests, whisker movement was not significantly different between Compression and Sham groups 2 weeks post injury (*p* > 0.05), indicating recovery of facial nerve function following compression. In contrast, the Axotomy group continued to display significantly lower scores than the Sham group at 3 weeks (F = 465.043, *p* < 0.001), 4 weeks (F = 392.413, *p* < 0.001), 8 weeks (F = 352, *p* < 0.001), and 12 weeks (F = 269.5, *p* < 0.001) after facial nerve injury.

Scores from the eyelid blink-reflex test (Figure 1B) were significantly lower in Compression and Axotomy groups compared with the Sham group at 1 week (F = 61.727, *p* < 0.001) post injury. At 2 weeks post injury, no significant difference was observed between Compression and Sham groups, indicating that facial nerve function had recovered following compression. In contrast, the Axotomy group consistently showed significantly lower scores than the Sham group at 2 weeks (F = 53.233, *p* < 0.001), 3 weeks (F = 30.164, *p* < 0.001), 4 weeks (F = 114.333, *p* < 0.001), 8 weeks (F = 40, *p* < 0.001), and 12 weeks (F = 45, *p* < 0.001) after facial nerve injury.

### 2.2. Changes in SMAD1–8 Protein Expression Following Facial Nerve Injury Induced by Compression or Axotomy

**Figure 2:**
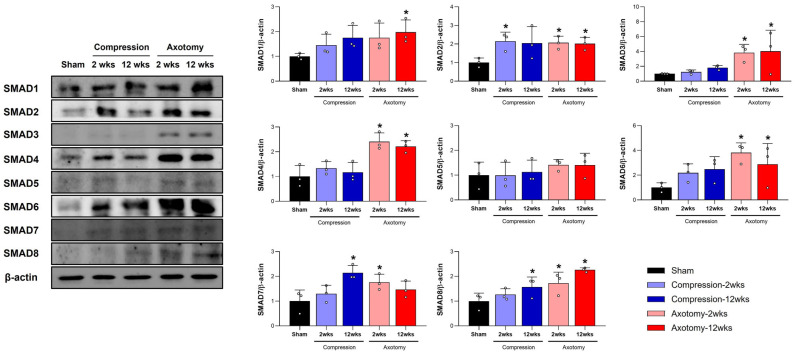
Differential expression of SMAD1–8 proteins in the peripheral facial nerve following injury. Western blot analysis of SMAD1–8 protein levels in the peripheral facial nerve at 2 and 12 weeks post injury induced by compression or axotomy. At 2 weeks, compression injury led to a significant increase in SMAD2 expression. By 12 weeks, SMAD7 and SMAD8 expression were elevated in the Compression group. In contrast, axotomy resulted in significant upregulation of SMAD1, SMAD2, SMAD3, SMAD4, SMAD6, SMAD7, and SMAD8 at both 2 and 12 weeks; no change in SMAD5 expression was observed in either injury model. The complete gel images are available in Supplementary Figures S1 and S2 for reference. Data are presented as means ± S.D., with a sample size of *n* = 3 per group (* *p* < 0.05 vs. Sham group). Abbreviations: wks, weeks.

Figure 2 shows the expression of SMAD1–8 proteins in the peripheral facial nerve at 2 and 12 weeks following facial nerve injury induced by compression or axotomy. Two weeks after a compressive facial nerve injury, the expression of SMAD2 increased in the Compression group compared to the Sham group (F = 10.844, *p* < 0.01); by 12 weeks after facial nerve injury, the expression of SMAD7 (F = 19.421, *p* < 0.001) and SMAD8 (F = 5.786, *p* < 0.05) had increased in the Compression group. In contrast, 2 weeks after injury by axotomy, the expression of SMAD1 (F = 3.391, *p* < 0.05), SMAD2 (F = 11.012, *p* < 0.01), SMAD3 (F = 6.966, *p* < 0.01), SMAD4 (F = 34.481, *p* < 0.001), SMAD6 (F = 12.88, *p* < 0.001), SMAD7 (F = 7.837, *p* < 0.01), and SMAD8 (F = 29.282, *p* < 0.001) increased in the Axotomy group compared with the Sham group, and this increase persisted at 12 weeks. The expression of SMAD5 (F = 1.564, *p* = 0.215) remained unchanged in the peripheral facial nerve after facial nerve injury by either compression or axotomy.

## 3. Materials and Methods

### 3.1. Animals

Male Sprague-Dawley rats weighing 200–250 g at 6 weeks of age (Orient Bio, Seong-nam, Gyeonggi-do, Republic of Korea) were used. Rats were housed in a controlled environment at 22 ± 2 °C with 50% humidity and maintained on a 12-h light/dark cycle with ad libitum access to food and water. The study protocol was approved by the Clinical Research Ethics Committee of Kyung Hee University Medical Center (KHMC-IACUC 22-044). During the 1-week acclimatization period, rats were handled daily to minimize stress and acclimate them to the researchers. After acclimatization, the rats were randomly assigned to Compression (*n* = 12), Axotomy (*n* = 12), or Sham (*n* = 6) groups, with health and welfare monitored regularly throughout the study.

### 3.2. Compression and Axotomy Procedures for Facial Nerve Injury

Before initiating facial nerve injury procedures (compression or axotomy) in rats, anesthesia was administered using 5% isoflurane (Foran solution; Hwaseong-si, Republic of Korea) in a mixture of 80% oxygen. This was maintained at 3% isoflurane throughout the procedure. Under general anesthesia, the left retroauricular area was prepared by disinfecting with 70% alcohol and shaving to clearly reveal the surgical site. A precise incision was made in this area to access the parotid gland and mastoid process, allowing for careful identification of the facial nerve trunk and its five branches: temporal, zygomatic, buccal, mandibular, and cervical. In the Compression group, the facial nerve trunk was subjected to a 30-s compression using fine forceps. For the Axotomy group, a 5 mm segment of the facial nerve trunk was fully transected. Post-procedure, the incision was sutured to close the surgical site. Following surgery, the rats were monitored during a 3-day recovery period in a warm, comfortable environment. During this time, their body weight, behavior, and any potential wound complications were closely observed. In the Sham group, only a skin incision in the left retroauricular region was performed, without applying compression or axotomy, and the incision was subsequently sutured under inhalation anesthesia.

### 3.3. Behavioral Tests

To evaluate the extent of damage and the subsequent recovery process following facial nerve injury, we implemented a series of behavioral assessments. These assessments focused on two key indicators of facial nerve function: whisker movement and the eyelid blink reflex. Observations were conducted at multiple time points post-injury, specifically at 1, 2, 3, 4, 8, and 12 weeks. The whisker movement test was designed to assess the functionality of the vibrissae muscle by closely observing the motion of the whiskers. During this test, each rat was gently restrained with both hands. This gentle restraint minimized stress and movement, allowing for accurate and consistent observations. We used the Vibrissae Observation Scale, a five-point system, to score the extent of whisker movement on the injured side. A score of 5 indicated normal whisker movement, where the whiskers on the injured side moved forward in a manner similar to those on the uninjured side. A score of 4 was assigned when movement was normal but accompanied by a noticeable backward tilt. A score of 3 represented significant movement, albeit with a backward tilt. A score of 2 indicated slight movement, with the whiskers remaining laid back, and a score of 1 represented no movement at all, with the whiskers staying laid back. For the eyelid blink-reflex test, we evaluated the eyelid’s response to external stimuli. This was done by delivering air puffs of consistent intensity to the area surrounding the eyes, using a calibrated air pump. The degree of eyelid closure was assessed using the Eye Closing and Blinking Reflex Observation Scale. A score of 5 indicated complete eyelid closure, representing a normal blink reflex. A score of 4 represented 75% closure, while a score of 3 indicated 50% closure. A score of 2 was given for contraction without achieving full closure, and a score of 1 indicated no eyelid movement. These behavioral tests provided valuable insights into the functional recovery of facial nerve activity.

### 3.4. Tissue Preparation

At 2 and 12 weeks post-injury, rats were euthanized to collect facial nerve tissues. The tissues were rinsed in cold PBS, flash-frozen with liquid nitrogen, and stored at −80 °C. For protein extraction, the samples were homogenized in RIPA buffer. This buffer was supplemented with a protease and phosphatase inhibitor cocktail (Thermo Fisher Scientific, 168 Third Avenue, Waltham, MA, USA) to maintain the integrity and activity of the proteins within the samples. The homogenization process was followed by centrifugation at 12,000× *g* for 15 min at 4 °C, which facilitated the separation of cellular debris from the protein-rich supernatant. The supernatants, now containing solubilized proteins, were carefully collected. To quantify the protein concentration, we employed the bicinchoninic acid (BCA) protein assay kit (Thermo Fisher Scientific, 168 Third Avenue, Waltham, MA, USA), which is known for its accuracy and reliability in protein quantification. Once quantified, the protein extracts were divided into aliquots and stored at −80 °C, ensuring they remained stable and ready for subsequent Western blot analysis.

### 3.5. Western Blotting

To analyze protein expression, 25 µg of protein from clarified lysate supernatants was separated using 8–10% SDS-PAGE gels. Proteins were transferred onto PVDF membranes, which were then blocked with 5% nonfat milk in TBS to prevent nonspecific binding. Membranes were incubated overnight at 4 °C with primary antibodies for specific proteins, including SMAD proteins and β-actin as a loading control. The antibodies used were as follows: SMAD1 (Santa Cruz, 10410 Finnell Street, Dallas, TX 75220, USA, sc-7965, 1:1000), SMAD2 (Santa Cruz, sc-7965, 1:500), SMAD3 (Cell Signaling Technology, 3 Trask Lane, Danvers, MA 01923, USA, 38454S, 1:500), SMAD4 (Cell Signaling Technology, 9513S, 1:1000), SMAD5 (Cell Signaling Technology, 9517S, 1:500), SMAD6 (Novus, 10730 East Briarwood Avenue, Centennial, CO 80112, USA), NB100-56440, 1:1000), SMAD7 (R&D Systems, 614 McKinley Place NE, Minneapolis, MN 55413, USA), MAB2029, 1:500), SMAD8 (Santa Cruz, sc-518051, 1:500), and β-actin (Santa Cruz, sc-47778, 1:50,000). Following the primary antibody incubation, the membranes were thoroughly washed to remove unbound antibodies. They were then incubated with a secondary antibody, specifically a horseradish peroxidase-conjugated mouse anti-rabbit antibody, at a 1:5000 dilution for 2 h at room temperature. This secondary antibody facilitates the detection of the protein-antibody complexes. Protein bands were visualized using enhanced chemiluminescence (ECL) reagents (Clarity Western ECL Substrate; Bio-Rad, 1000 Alfred Nobel Drive, Hercules, CA 94547, USA), which produce a luminescent signal upon reaction with the horseradish peroxidase enzyme. The intensity of the luminescent signal was captured and quantified using Image J software 1.54 (United States National Institutes of Health, Bethesda, MD, USA) allowing for the analysis of protein expression levels.

### 3.6. Statistical Analysis

The data obtained from the experiments are presented as the means of at least two replicates and are expressed as means ± standard error of the mean (S.E.M.). To analyze the data, statistical tests were conducted using SPSS software (version 25; IBM SPSS Corp., Armonk, NY, USA). Experimental results were compared using one-way analysis of variance (ANOVA), followed by post hoc Tukey test for group comparisons. A significance level of *p* < 0.05 was considered to indicate statistically significant differences.

## 4. Discussion

In this study, we examined SMAD1–8 protein expression in rat models of peripheral facial nerve injury, focusing on compression and axotomy injuries. Our findings reveal distinct SMAD protein expression profiles, suggesting different roles in nerve regeneration. Behavioral assessments showed significant facial nerve impairment at early stages for both injury types. However, the Compression group recovered by 3 weeks, while the Axotomy group showed prolonged impairment up to 12 weeks, highlighting differences in regenerative capacity. SMAD protein analysis indicated that compression injury increased SMAD2, SMAD7, and SMAD8 expression, potentially facilitating quicker recovery. Axotomy led to broader SMAD upregulation, including SMAD1, SMAD2, SMAD3, SMAD4, SMAD6, SMAD7, and SMAD8, suggesting a complex repair mechanism possibly causing delayed recovery. The differential SMAD expression illustrates the complex molecular pathways in nerve regeneration, with potential therapeutic targets for enhancing nerve repair. Enhancing beneficial SMAD protein expression could promote recovery in severe nerve injuries. These insights deepen our understanding of molecular responses to peripheral nerve injuries and underscore the potential of targeting specific SMAD proteins for improved therapeutic outcomes.

In this study, we examined SMAD1–8 protein expression in response to facial nerve injuries caused by compression and axotomy in rats. Our behavioral and molecular data revealed distinct recovery patterns and SMAD protein involvement between these two injury models. Compression injuries, which typically cause local demyelination and mild axonal damage, showed functional impairments lasting up to 2 weeks, with recovery by 3 weeks, consistent with previous studies [17,18]. This suggests that compression damage, though initially significant, tends to resolve naturally. Conversely, axotomy, involving complete nerve transection, serves as a model for nerve regeneration, resulting in prolonged impairment with no recovery even after 12 weeks [17]. This difference in recovery timelines underscores the severity and complexity of nerve regeneration following different injury types.

SMAD proteins are central mediators of the TGF-β signaling pathway, playing a critical role in various cellular processes, including cell growth, differentiation, and repair mechanisms [19]. Within this framework, different SMAD proteins serve distinct functions: receptor-regulated SMADs (R-SMADs), such as SMAD1, SMAD2, SMAD3, SMAD4, and SMAD8, typically propagate signals, whereas inhibitory SMADs, like SMAD6 and SMAD7, act to counterbalance these signals. This balance is crucial in determining the outcome of nerve regeneration, as it can either promote or inhibit the regenerative process depending on the specific SMAD protein involved and the context of the injury.

SMAD1 is activated by BMP ligand binding to BMP receptors [20]. Once phosphorylated, it forms a complex with SMAD4 and moves to the nucleus to regulate gene expression. SMAD1 enhances neuronal growth capacity by regulating genes for axonal regeneration [21] and modulates the inflammatory response to support nerve repair. Activation of BMP4/SMAD1 signaling enhances axon regeneration and functional recovery in the spinal dorsal column [22], and improves survival and regeneration of retinal ganglion cells after optic nerve injury [23]. Our study showed increased SMAD1 protein levels following facial nerve axotomy, indicating a sustained role in regeneration or repair, consistent with its involvement in BMP signaling, crucial for cellular repair mechanisms.

SMAD2 is activated by TGF-β ligand binding to its receptor [24]. Once phosphorylated, it forms a complex with SMAD4 and translocates to the nucleus to regulate gene expression [25]. SMAD2 influences the inflammatory response after nerve injury by reducing excessive inflammation, which can hinder regeneration. It regulates fibrosis by modulating fibroblast activity, preventing scar tissue formation that could obstruct nerve regeneration, and shapes the environment around the injury site to support repair. In a sciatic nerve compression injury model, SMAD2 signaling enhanced axonal regeneration through interactions with cholesterol metabolism [26]. Our study found increased SMAD2 protein levels following facial nerve injury, suggesting its role in nerve regeneration.

SMAD3 plays a crucial role in the initial inflammatory response, a key step in initiating repair processes [27]. As a mediator of the TGF-β signaling pathway, SMAD3 has complex roles in inflammation and fibrosis following nerve injury [14]. If unregulated, it can lead to excessive fibrosis, hindering regeneration. In traumatic brain injury, SMAD3 protects neurons in regions like the hippocampus and cortex, with its deficiency causing increased neuronal loss [28], highlighting its protective role. Our study shows increased SMAD3 levels after facial nerve amputation, indicating its involvement in regeneration. In spinal cord injury models, however, inhibiting SMAD3 reduced neuronal death and inflammation by preventing caspase-1-induced pyroptosis [29]. These findings suggest that SMAD3’s effects are context-specific, with its modulation potentially promoting regeneration or exacerbating damage, depending on the injury type and cellular environment.

SMAD4 serves as a central mediator in the TGF-β signaling pathway, acting in conjunction with receptor-regulated SMADs to regulate the expression of genes critical for nerve repair [30]. SMAD4 mRNA levels were found to be upregulated following compression-induced spinal cord injury [14]. This underscores SMAD4’s role in orchestrating the cellular responses needed for effective nerve repair and regeneration after injury. In facial nerve injuries, whether from compression or axotomy, SMAD4 facilitates axonal growth and nerve regeneration by promoting the transcription of genes related to cell survival, growth, and differentiation. Our study further supports this regenerative role, as we observed that increased levels of SMAD4 were associated with enhanced nerve regeneration following facial nerve injury. This suggests that SMAD4 is integral to the recovery and repair processes, highlighting its potential as a therapeutic target for improving outcomes in nerve injury treatments.

A point of emphasis for SMAD5 is its essential function within the BMP pathway, a critical signaling axis regulated by members of the TGF-β superfamily [31]. SMAD5 acts as an intracellular mediator that transmits signals from BMP receptors on the cell surface to the nucleus, influencing the transcription of genes involved in cell proliferation, differentiation, and apoptosis. The expression levels of SMAD5 mRNA are not reported to significantly change following nerve injury [32]. This suggests that, at the mRNA level, SMAD5 may not play a prominent or direct role in the neuronal response to injury under the specific experimental conditions examined. The absence of changes in mRNA expression does not necessarily rule out SMAD5 involvement in the injury response; however, we also found no changes in SMAD5 protein levels following facial nerve injury in the present study.

SMAD6 inhibits the phosphorylation and activation of receptor-regulated SMADs, modulating both BMP and TGF-β signaling pathways [33]. As an inhibitory SMAD, it plays a key role in regulating BMP signaling. Its regulation is linked to excessive or maladaptive repair processes that may impede nerve regeneration. By blocking signal transduction [34], SMAD6 acts as an inhibitor in nerve regeneration following injury. It is a negative regulator of the TGF-β pathway, controlling processes like proliferation and differentiation [35]. Although SMAD6 does not directly interact with SMAD2 or SMAD4, it inhibits the SMAD1-SMAD4 complex formation, acting as an antagonist of the BMP/SMAD1 pathway and potentially inhibiting nerve regeneration [34]. Our study found SMAD6 expression was upregulated after facial nerve injury. Modulating SMAD6 activity could enhance nerve regeneration or prevent adverse outcomes post-injury.

SMAD7 is an inhibitory SMAD protein known for blocking TGF-β signaling; its overexpression can increase inflammation [36]. Inhibiting SMAD7 reduces inflammatory cytokine production and inflammation in chronic inflammatory bowel disease [37], highlighting its role in modulating inflammation. In nerve injury, SMAD7’s role is complex. Co-overexpression with VEGF has been shown to improve outcomes post-injury [38], indicating that SMAD7 may interact with VEGF and TGF-β pathways to promote angiogenesis and nerve repair. Our study found increased SMAD7 levels following facial nerve injury, suggesting its dual role as a regulatory node in inflammation and regeneration. Further research is needed to understand when SMAD7 shifts from an inflammatory modulator to a regenerative facilitator, which could be crucial for developing targeted nerve injury therapies.

SMAD8 is an intracellular signaling molecule that transduces signals from the TGF-β superfamily [39]. After nerve injury, SMAD8 mRNA levels decrease in motor neurons, suggesting a potentially different or inhibitory role in nerve repair compared to other upregulated SMADs like SMAD1, SMAD2, and SMAD4 [32]. In Duchenne muscular dystrophy, the BMP4/SMAD8 pathway is upregulated alongside IL-6, a key inflammatory mediator [40]. This pathway regulates tissue repair and wound healing. The absence of SMAD8 in hepatocytes leads to dysregulated iron metabolism and liver fibrosis [41], indicating that SMAD8 may play a compensatory role in repair and regeneration by maintaining normal metabolic processes.

Our study observed increased SMAD8 protein levels following facial nerve injury, suggesting its role in supporting nerve repair and regeneration. The BMP-SMAD1/5/8 pathway is crucial for maintaining signaling balance for neuronal development and regeneration [16]. After sciatic nerve injury, SMAD1, SMAD5, and SMAD8 levels rise significantly in the gastrocnemius muscle, indicating their active involvement in muscle response to nerve injury. This upregulation and enhanced phosphorylation imply a role in regeneration or muscle tissue remodeling [42]. Receptors for BMPs and p-SMAD1/5/8 were notably upregulated a week after sciatic nerve injury [43]. Our findings of increased SMAD1 and SMAD8 levels following facial nerve injury suggest their regulation is linked to nerve regeneration, potentially aiding in developing therapeutic strategies.

In Supplementary Figure S3, the scatter plots illustrate the correlation between behavioral experiments and SMAD1–8 expression levels. A correlation value close to 1 suggests a strong relationship. For example, in the vibrissae response, SMAD3, 4, and 6 show a high correlation, suggesting a significant relationship. Similarly, in the eye-closing tests, SMAD3, 4, 6, 7, and 8 display high correlations, indicating strong associations. Overall, the results from the vibrissae reflex and eye-closing tests in facial nerve injury, alongside changes in SMAD1–8 proteins, suggest a significant correlation between facial nerve injury and both vibrissae and eye-blink reflex outcomes.

## 5. Conclusions

Our study provides valuable insights into the differential expression and roles of SMAD proteins in compression and axotomy peripheral facial nerve injury models. We observed distinct expression patterns of SMAD1–8 proteins, indicating that these proteins have specific and context-dependent roles in nerve regeneration processes. Notably, compression injury resulted in the upregulation of SMAD2, SMAD7, and SMAD8, whereas axotomy led to a broader increase in several SMAD proteins, including SMAD1, SMAD2, SMAD3, SMAD4, SMAD6, SMAD7, and SMAD8. These findings underscore the complexity of the molecular mechanisms underlying nerve regeneration and suggest that different types of nerve injuries activate unique SMAD-mediated pathways. While our findings suggest potential avenues for therapeutic exploration, they do not, on their own, establish therapeutic value. The differential involvement of specific SMAD proteins in these pathways highlights their potential as therapeutic targets for enhancing recovery after peripheral nerve injuries. However, the balance between receptor-regulated SMADs and inhibitory SMADs appears crucial for determining the outcome of nerve regeneration, which could either facilitate or hinder the regenerative process depending on the injury context and specific SMAD proteins involved. Overall, our study suggests that modulating SMAD protein activity could offer novel therapeutic strategies for improving outcomes in nerve injury treatments. However, future functional studies are necessary to substantiate any therapeutic implications of SMAD upregulation. Further research should focus on elucidating the precise molecular interactions and signaling pathways involving SMAD proteins to develop targeted interventions for nerve repair and regeneration.

## Data Availability

Data are contained within the article.

## Supplementary Materials

The following supporting information can be downloaded at: www.mdpi.com/article/10.3390/ijms26052291/s1.

## References

[1] Baugh R.F., Basura G.J., Ishii L.E., Schwartz S.R., Drumheller C.M., Burkholder R., Deckard N.A., Dawson C., Driscoll C., Gillespie M.B. (2013). Clinical Practice Guideline: Bell’s Palsy. Otolaryngol. Head. Neck Surg..

[2] Finsterer J. (2008). Management of Peripheral Facial Nerve Palsy. Eur. Arch. Oto-Rhino-Laryngol..

[3] Greco A., Gallo A., Fusconi M., Marinelli C., Macri G.F., de Vincentiis M. (2012). Bell’s Palsy and Autoimmunity. Autoimmun. Rev..

[4] Stierli S., Napoli I., White I.J., Cattin A.-L., Monteza Cabrejos A., Garcia Calavia N., Malong L., Ribeiro S., Nihouarn J., Williams R. (2018). The Regulation of the Homeostasis and Regeneration of Peripheral Nerve Is Distinct from the CNS and Independent of a Stem Cell Population. Development.

[5] Menorca R.M.G., Fussell T.S., Elfar J.C. (2013). Nerve Physiology. Hand Clin..

[6] Sulaiman W., Gordon T. (2013). Neurobiology of Peripheral Nerve Injury, Regeneration, and Functional Recovery: From Bench Top Research to Bedside Application. Ochsner J..

[7] Jessen K.R., Mirsky R. (2016). The Repair Schwann Cell and Its Function in Regenerating Nerves. J. Physiol..

[8] Stoll G., Müller H.W. (1999). Nerve Injury, Axonal Degeneration and Neural Regeneration: Basic Insights. Brain Pathol..

[9] van Niekerk E.A., Tuszynski M.H., Lu P., Dulin J.N. (2016). Molecular and Cellular Mechanisms of Axonal Regeneration After Spinal Cord Injury. Mol. Cell. Proteom..

[10] Lopes B., Sousa P., Alvites R., Branquinho M., Sousa A.C., Mendonça C., Atayde L.M., Luís A.L., Varejão A.S.P., Maurício A.C. (2022). Peripheral Nerve Injury Treatments and Advances: One Health Perspective. Int. J. Mol. Sci..

[11] Coutinho A.E., Chapman K.E. (2011). The Anti-Inflammatory and Immunosuppressive Effects of Glucocorticoids, Recent Developments and Mechanistic Insights. Mol. Cell Endocrinol..

[12] Grinsell D., Keating C.P. (2014). Peripheral Nerve Reconstruction after Injury: A Review of Clinical and Experimental Therapies. Biomed. Res. Int..

[13] Wei C., Guo Y., Ci Z., Li M., Zhang Y., Zhou Y. (2024). Advances of Schwann Cells in Peripheral Nerve Regeneration: From Mechanism to Cell Therapy. Biomed. Pharmacother..

[14] Lee J., Yon D.K., Choi Y.S., Lee J., Yeo J.H., Kim S.S., Lee J.M., Yeo S.G. (2024). Roles of SMAD and SMAD-Associated Signaling Pathways in Nerve Regeneration Following Peripheral Nerve Injury: A Narrative Literature Review. Curr. Issues Mol. Biol..

[15] Zhang Y., Alexander P.B., Wang X.-F. (2017). TGF-β Family Signaling in the Control of Cell Proliferation and Survival. Cold Spring Harb. Perspect. Biol..

[16] Hegarty S.V., O’Keeffe G.W., Sullivan A.M. (2013). BMP-Smad 1/5/8 Signalling in the Development of the Nervous System. Prog. Neurobiol..

[17] Lee J.M., Yoo M.C., Kim Y.J., Kim S.S., Yeo S.G. (2024). Expression of ChAT, Iba-1, and NNOS in the Central Nervous System Following Facial Nerve Injury. Antioxidants.

[18] Kim I., Kim Y., Kang D., Jung J., Kim S., Rim H., Kim S., Yeo S.-G. (2021). Neuropeptides Involved in Facial Nerve Regeneration. Biomedicines.

[19] Hata A., Chen Y.-G. (2016). TGF-β Signaling from Receptors to Smads. Cold Spring Harb. Perspect. Biol..

[20] Hassel S., Schmitt S., Hartung A., Roth M., Nohe A., Petersen N., Ehrlich M., Henis Y.I., Sebald W., Knaus P. (2003). Initiation of Smad-Dependent and Smad-Independent Signaling via Distinct BMP-Receptor Complexes. J. Bone Jt. Surg. Am..

[21] Zou H., Ho C., Wong K., Tessier-Lavigne M. (2009). Axotomy-Induced Smad1 Activation Promotes Axonal Growth in Adult Sensory Neurons. J. Neurosci..

[22] Farrukh F., Davies E., Berry M., Logan A., Ahmed Z. (2019). BMP4/Smad1 Signalling Promotes Spinal Dorsal Column Axon Regeneration and Functional Recovery After Injury. Mol. Neurobiol..

[23] Thompson A., Berry M., Logan A., Ahmed Z. (2019). Activation of the BMP4/Smad1 Pathway Promotes Retinal Ganglion Cell Survival and Axon Regeneration. Investig. Opthalmology Vis. Sci..

[24] Shi Y., Massagué J. (2003). Mechanisms of TGF-β Signaling from Cell Membrane to the Nucleus. Cell.

[25] Liu L., Liu X., Ren X., Tian Y., Chen Z., Xu X., Du Y., Jiang C., Fang Y., Liu Z. (2016). Smad2 and Smad3 Have Differential Sensitivity in Relaying TGFβ Signaling and Inversely Regulate Early Lineage Specification. Sci. Rep..

[26] Lee J., Shin J.E., Lee B., Kim H., Jeon Y., Ahn S.H., Chi S.W., Cho Y. (2020). The Stem Cell Marker *Prom1* Promotes Axon Regeneration by down-Regulating Cholesterol Synthesis via Smad Signaling. Proc. Natl. Acad. Sci. USA.

[27] Tan N.S., Michalik L., Di-Poï N., Ng C.Y., Mermod N., Roberts A.B., Desvergne B., Wahli W. (2004). Essential Role of Smad3 in the Inhibition of Inflammation-Induced PPARβ/δ Expression. EMBO J..

[28] Villapol S., Wang Y., Adams M., Symes A.J. (2013). Smad3 Deficiency Increases Cortical and Hippocampal Neuronal Loss Following Traumatic Brain Injury. Exp. Neurol..

[29] Zhu J., Fu Y., Tu G. (2020). Role of Smad3 Inhibitor and the Pyroptosis Pathway in Spinal Cord Injury. Exp. Ther. Med..

[30] Datto M., Wang X.-F. (2000). The Smads: Transcriptional Regulation and Mouse Models. Cytokine Growth Factor Rev..

[31] Chang H., Matzuk M.M. (2001). Smad5 Is Required for Mouse Primordial Germ Cell Development. Mech. Dev..

[32] Okuyama N., Kiryu-Seo S., Kiyama H. (2007). Altered Expression of Smad Family Members in Injured Motor Neurons of Rat. Brain Res..

[33] Miyazawa K., Miyazono K. (2017). Regulation of TGF-β Family Signaling by Inhibitory Smads. Cold Spring Harb. Perspect. Biol..

[34] Hata A., Lagna G., Massagué J., Hemmati-Brivanlou A. (1998). Smad6 Inhibits BMP/Smad1 Signaling by Specifically Competing with the Smad4 Tumor Suppressor. Genes. Dev..

[35] Gélabert C., Papoutsoglou P., Golán I., Ahlström E., Ameur A., Heldin C.-H., Caja L., Moustakas A. (2023). The Long Non-Coding RNA LINC00707 Interacts with Smad Proteins to Regulate TGFβ Signaling and Cancer Cell Invasion. Cell Commun. Signal..

[36] Hu Y., He J., He L., Xu B., Wang Q. (2021). Expression and Function of Smad7 in Autoimmune and Inflammatory Diseases. J. Mol. Med..

[37] Monteleone G., Kumberova A., Croft N.M., McKenzie C., Steer H.W., MacDonald T.T. (2001). Blocking Smad7 Restores TGF-Β1 Signaling in Chronic Inflammatory Bowel Disease. J. Clin. Investig..

[38] He L., Yu T., Xiao Y., Huang Y., Guan Y., Zhao F., Ma L. (2022). Co-Overexpression of VEGF and Smad7 Improved the Therapeutic Effects of Adipose-Derived Stem Cells on Neurogenic Erectile Dysfunction in the Rat Model. Andrologia.

[39] Chen Y., Bhushan A., Vale W. (1997). Smad8 Mediates the Signaling of the Receptor Serine Kinase. Proc. Natl. Acad. Sci. USA.

[40] Lopez M.A., Si Y., Hu X., Williams V., Qushair F., Carlyle J., Alesce L., Conklin M., Gilbert S., Bamman M.M. (2022). Smad8 Is Increased in Duchenne Muscular Dystrophy and Suppresses MiR-1, MiR-133a, and MiR-133b. Int. J. Mol. Sci..

[41] Wang C., Xiao X., Bayer A., Xu Y., Dev S., Canali S., Nair A.V., Masia R., Babitt J.L. (2019). Ablation of Hepatocyte Smad1, Smad5, and Smad8 Causes Severe Tissue Iron Loading and Liver Fibrosis in Mice. Hepatology.

[42] Si Y., Cui X., Kim S., Wians R., Sorge R., Oh S.J., Kwan T., AlSharabati M., Lu L., Claussen G. (2014). Smads as Muscle Biomarkers in Amyotrophic Lateral Sclerosis. Ann. Clin. Transl. Neurol..

[43] Kokubu N., Tsujii M., Akeda K., Iino T., Sudo A. (2018). BMP-7/Smad Expression in Dedifferentiated Schwann Cells during Axonal Regeneration and Upregulation of Endogenous BMP-7 Following Administration of PTH (1–34). J. Orthop. Surg..

