# Supplementary material for: Expression Patterns of SMAD1–8 in the Peripheral Facial Nerve Following Compressive Nerve Injury or Axotomy
Source: Int J Mol Sci. 2025 Mar 4;26(5):2291. doi: 10.3390/ijms26052291 (PMC11900376; doi:10.3390/ijms26052291)
Supplement: Supplementary file 1 [file ijms-26-02291-s001.zip › ijms-3440241-supplementary.pdf]

Contents

Figure S1. Full Gel Images for Three Experiments Corresponding to Figure 2 p. S2

Figure S2. Complete Gel Images Corresponding to Figure 2 p. S3-4

Figure S3. Scatter plot p. S5

Supplementary Figure S1. Full Gel Images for Three Experiments Corresponding to Figure 2

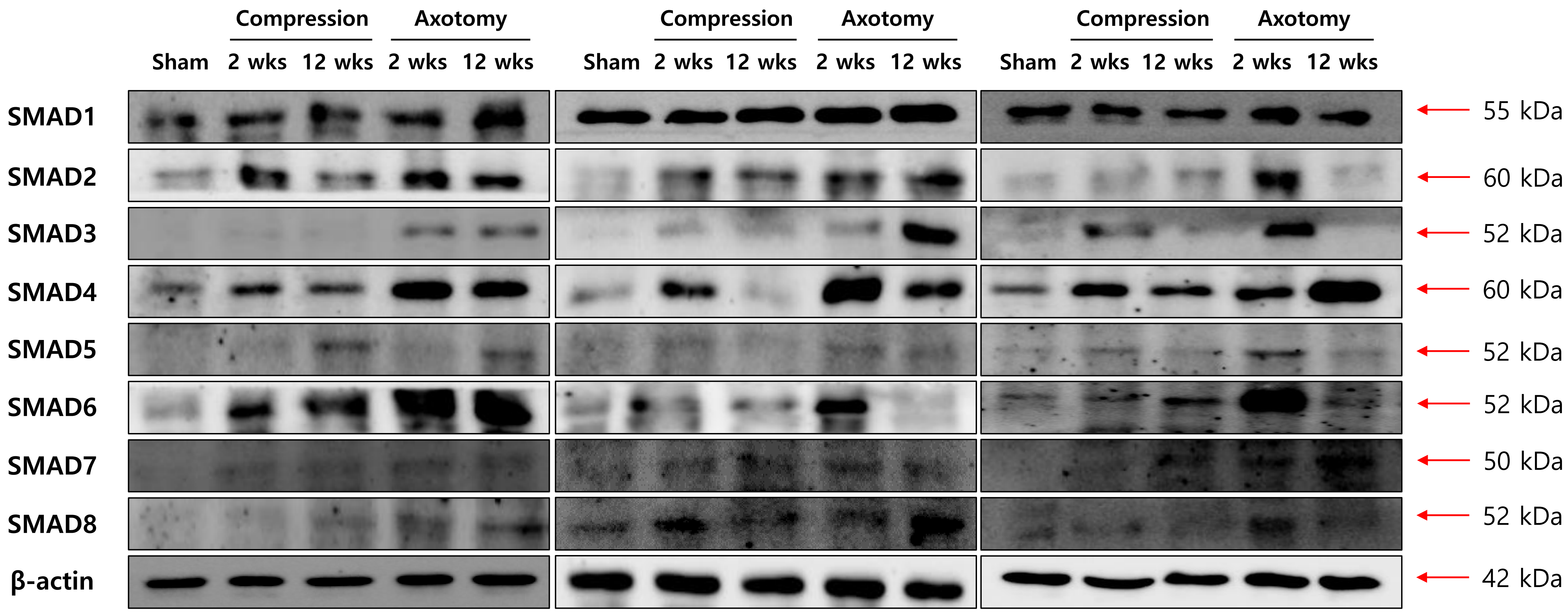

Figure S1. Full Gel Images for Three Experiments Corresponding to Figure 2. This figure displays the complete gel images from three independent experiments, each corresponding to the results shown in Figure 2.

Supplementary Figure S2. Complete Gel Images Corresponding to Figure 2

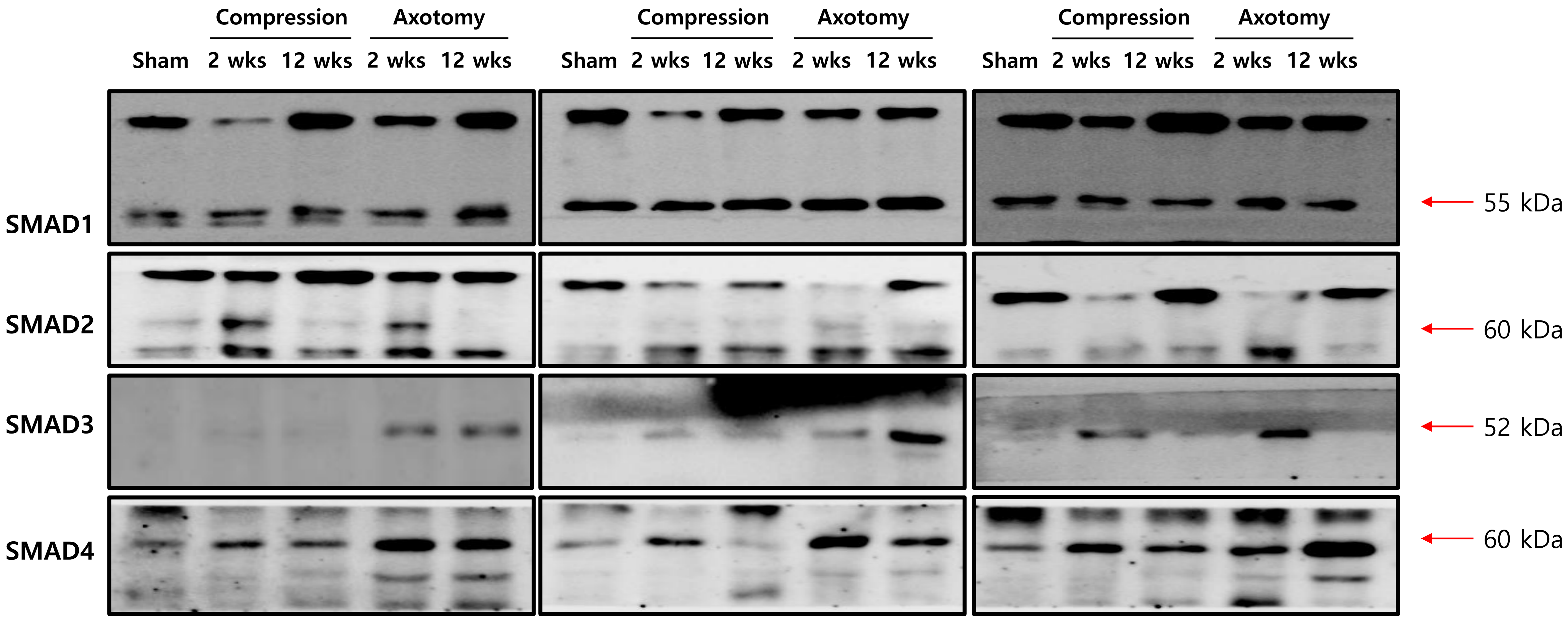

Figure S2. Complete Gel Images Corresponding to Figure 2. This supplementary figure presents the full gel images associated with the results shown in Figure 2

Supplementary Figure S2. Complete Gel Images Corresponding to Figure 2

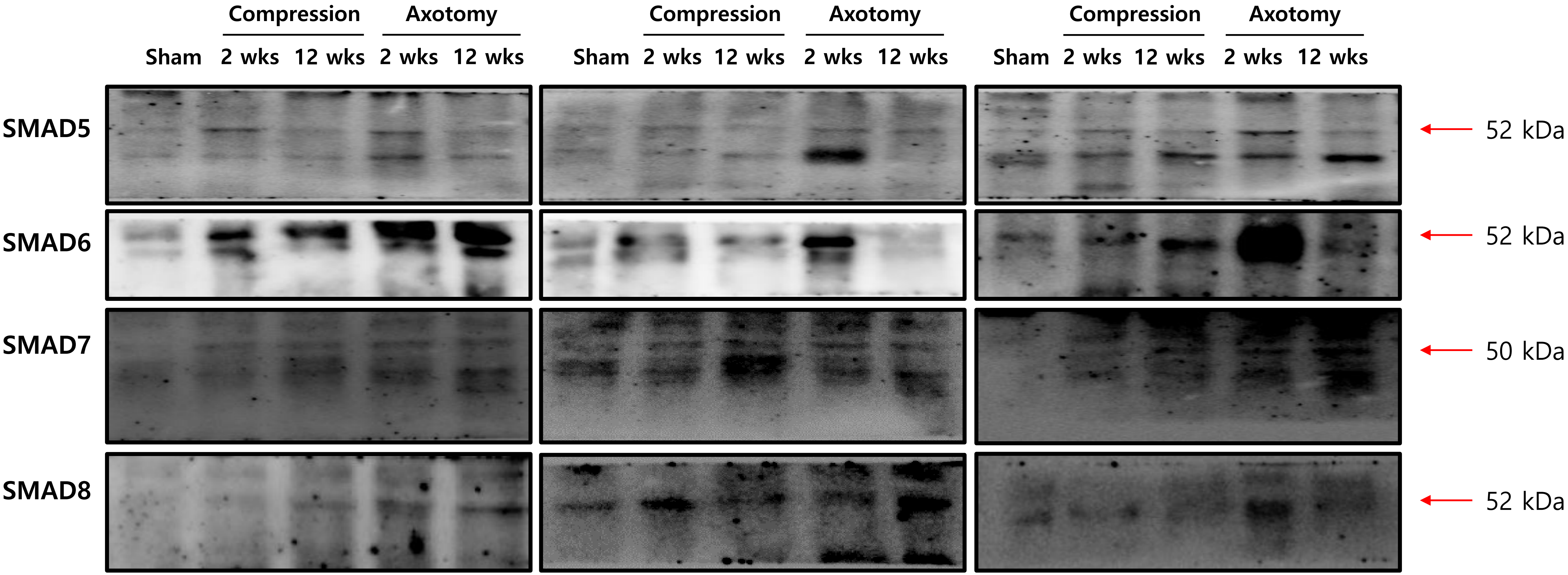

Figure S2. Complete Gel Images Corresponding to Figure 2. This supplementary figure presents the full gel images associated with the results shown in Figure 2

Supplementary Figure S3. Scatter plot

Correlations

Variable	Variable2	Correlation	Count	Lower C.I.	Upper C.I.	Notes
		Statistic				
Vibrissae_observation_scale	SMAD1	-.567	18	-.817	-.136	
	SMAD2	-.422	18	-.743	.055	
	SMAD3	-.906	18	-.965	-.761	
	SMAD4	-.862	18	-.947	-.661	
	SMAD5	-.495	18	-.781	-.036	
	SMAD6	-.838	18	-.938	-.609	
	SMAD7	-.668	18	-.865	-.292	
	SMAD8	-.670	18	-.866	-.296	

Missing value handling: PAIRWISE, EXCLUDE. C.I. Level: 95.0

Correlations

Variable	Variable2	Correlation	Count	Lower C.I.	Upper C.I.	Notes
		Statistic				
Eye_closing_scale	SMAD1	-.606	18	-.836	-.194	
	SMAD2	-.464	18	-.765	.003	
	SMAD3	-.908	18	-.966	-.766	
	SMAD4	-.833	18	-.936	-.599	
	SMAD5	-.547	18	-.808	-.107	
	SMAD6	-.810	18	-.926	-.551	
	SMAD7	-.712	18	-.885	-.367	
	SMAD8	-.700	18	-.880	-.347	

Missing value handling: PAIRWISE, EXCLUDE. C.I. Level: 95.0

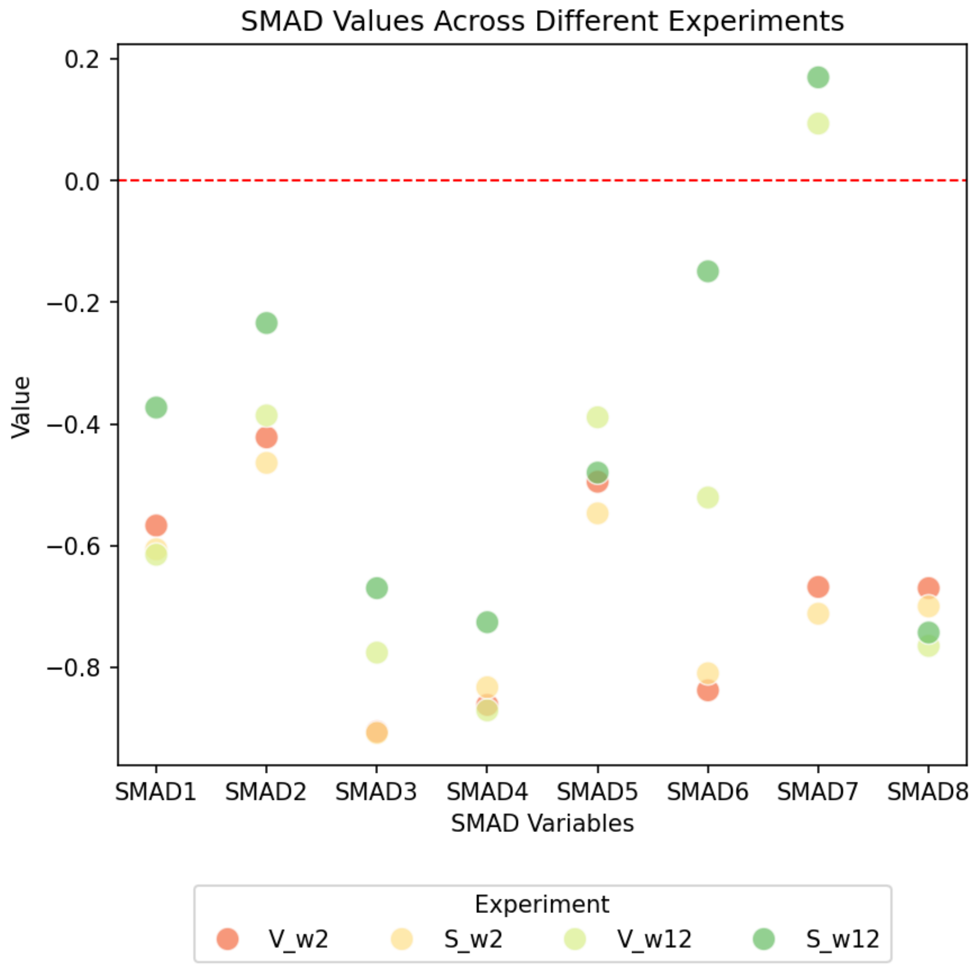

V; Vibrissae observation scale

S; Scale of eye closing and blinking reflex observation
